# Comprehensive validation of a wearable foot sensor system for estimating spatiotemporal gait parameters by simultaneous three-dimensional optical motion analysis

**DOI:** 10.1186/s13102-022-00461-x

**Published:** 2022-04-17

**Authors:** Kentaro Homan, Keizo Yamamoto, Ken Kadoya, Naoki Ishida, Norimasa Iwasaki

**Affiliations:** 1 Department of Orthopaedic Surgery, Faculty of Medicine and Graduate School of Medicine, Hokkaido University, Kita 15, Nishi 7, Kita-ku, Sapporo, Hokkaido 060-8638 Japan; 2 School of Lifelong Sport, Hokusho University, 23 Bunkyodai, Ebetsu, 069-8511 Japan; 3 Department of Orthopedic Surgery, Hokuto Medical Corporation Hokuto Hospital, Kisen 7-5 Inada-cho, Obihiro, Hokkaido Japan

**Keywords:** Wearable sensor, Validation study, Gait analysis, Three-dimensional motion analysis

## Abstract

**Background:**

Use of a wearable gait analysis system (WGAS) is becoming common when conducting gait analysis studies due to its versatility. At the same time, its versatility raises a concern about its accuracy, because its calculations rely on assumptions embedded in its algorithms. The purpose of the present study was to validate twenty spatiotemporal gait parameters calculated by the WGAS by comparison with simultaneous measurements taken with an optical motion capture system (OMCS).

**Methods:**

Ten young healthy volunteers wore two inertial sensors of the commercially available WGAS, Physilog®, on their feet and 23 markers for the OMCS on the lower part of the body. The participants performed at least three sets of 10-m walk tests at their self-paced speed in the laboratory equipped with 12 high-speed digital cameras with embedded force plates. To measure repeatability, all participants returned for a second day of testing within two weeks.

**Results:**

Twenty gait parameters calculated by the WGAS had a significant correlation with the ones determined by the OMCS. Bland and Altman analysis showed that the between-device agreement for twenty gait parameters was within clinically acceptable limits. The validity of the gait parameters generated by the WGAS was found to be excellent except for two parameters, swing width and maximal heel clearance. The repeatability of the WGAS was excellent when measured between sessions.

**Conclusion:**

The present study showed that spatiotemporal gait parameters estimated by the WGAS were reasonably accurate and repeatable in healthy young adults, providing a scientific basis for applying this system to clinical studies.

## Background

Gait analysis provides important information when diagnosing people with musculoskeletal and neurological disorders. Accumulated evidences indicate that the assessment of comprehensive gait characteristics can aid in making a diagnosis as well as planning a treatment more efficiently, compared to a simple measurement of gait speed [1–3]. Visual observation is the most common method, but it is subjective, not quantitative, and heavily dependent on the observer’s experience [4]. Although questionnaires or simple assessments about cadence, stance time, step length, and stride length help further our understanding of gait characteristics [5, 6], they are apparently insufficient to describe each gait characteristic, which is a highly complex set of movements of the whole body. To obtain an adequate understanding of gait characteristics, several instruments have been developed, and a combination of an optical motion capture system (OMCS) with force plates is currently considered the gold standard [7, 8]. Despite its great capacity to characterize gait, the OMCS remains uncommon in clinical practice due to its long operation time, dedicated space requirements and high cost.

As a result of the recent advances in information technology, new gait analysis systems have been developed [9, 10]. Inertial sensors combined with software incorporating special algorithms are now available as a wearable gait analysis system (WGAS) to measure spatiotemporal and kinematic gait parameters. Because the WGAS is usable anywhere inside and outside clinics and research laboratories, it can virtually evaluate unlimited numbers of gait cycles, and is less expensive than OMCSs, it has great versatility under a variety of conditions [11–13]. At the same time, the parameters calculated by WGASs depend totally on the assumptions embedded in their algorithms, raising potential concerns about accuracy and reliability [14]. Accordingly, an unusual gait or unexpected condition is likely to cause its calculations to be wrong. Clinical gait analysis targets various kinds of subjects from young to elderly persons, as well as various conditions from musculoskeletal to metabolic disorders, under different circumstances. Thus, it is critical to determine the range in which the WGAS can provide accurate estimates before conducting gait analysis research using this system. Although multiple gait parameters can be calculated simultaneously with the WGAS [15–31], no study comprehensively examined the actual accuracy of all of their gait parameters.

Physilog® (GaitUp, Lausanne, Switzerland) is one of the commercially available WGASs that can estimate 20 spatiotemporal gait parameters by only placing sensors on both feet. Accuracy of 18 parameters were partially verified by elemental methods using a prototype [22, 24, 32–35]. Two studies conducted validation of the current system in stroke patients, however, their analysis was limited to 7 parameters [22, 23]. Another study examined its reproducibility but not accuracy of 9 parameters in healthy subjects [25]. Therefore, the purpose of the present study was to validate spatiotemporal gait parameters calculated with Physilog® by comparing them to the same parameters measured simultaneously by the OMCS in healthy individuals. The present study provides a basis for clinical gait analysis using Physilog®, demonstrates a standard for other WGASs, and generates a rationale to use the WGASs for clinical gait analysis.

## Methods

### Participants

A total of 10 male volunteers (age 20.4 ± 0.5 years, weight 74.6 ± 5.2 kg, height 176.5 ± 3.7 cm, BMI 23.9 ± 1.5 kg/m^2^) with no signs of neurological or musculoskeletal impairment participated in the study. We recruited only males to eliminate the effects of gender variability on gait as previous WGSs validating studies [15, 27, 28, 36]. Exclusion criteria included: recent major ligament injury, surgery, fracture or muscle injury in the lower limb, abnormal gait pattern, contraindication to exercise, or other health conditions that would adversely impact gait characteristics. Based on previous validation studies with Physilog® [22, 23, 25], the sample size required to calculate the ICC was determined to be 10, which is the number of people required to satisfy the conditions of one examiner, with a significance level of 5%, an ICC estimate of 0.8, and a confidence interval of 0.2. All the 10 subjects had the right leg as dominant (the preferred limb used to kick a ball). The protocol for this study was approved by the Institutional Review Board of Hokkaido University (#16-062), and all participants provided their written, informed consent before participating.

### Walking protocol

**Fig. 1:**
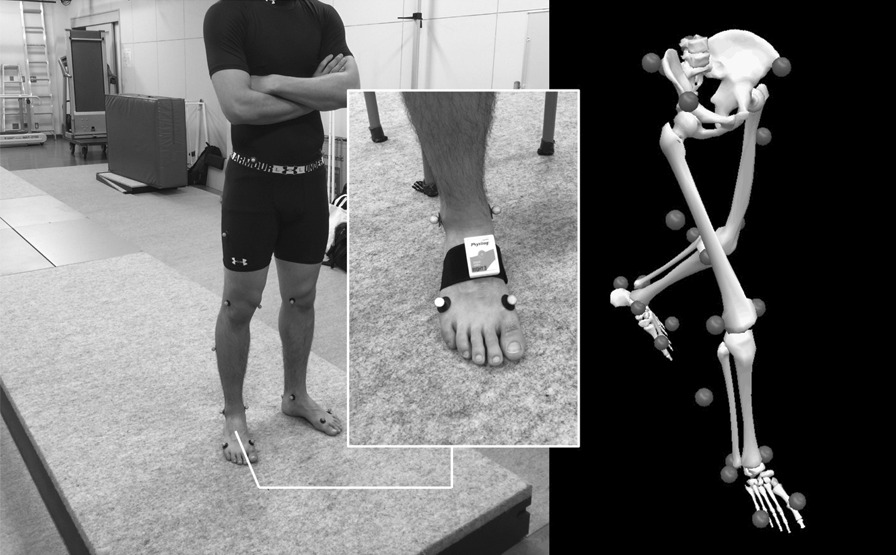
Left is a representative picture showing a subject with retro-reflective markers and Physilog® sensors on both feet in the room equipped for the OMCS. Right illustrates the positions of the retro-reflective markers for the OMCS

Subjects wore the Physilog® sensors (Physilog4) on each foot with a Velcro strap and the retro-reflective markers for the OMCS (Motion Analysis, Santa Rosa, USA) (Fig. 1). For simultaneous analysis, reflective markers were placed on both feet (first and fifth metatarsal joints, heels) and both ankles (medial and lateral condyles) of the participants with a foot configuration corresponding to the Physilog® sensors. The participants walked 10 round trips at a comfortable pace along a 10-m straight path, and 6 round trips in the middle of the session were used for the analysis. Four 0.5-m-long force plates (AMTI, Watertown, MA, USA) were embedded in the middle of the walkway to identify the time of heel-contact and toe-off. To identify each step on the force plate, each walk was recorded by a digital video camera. The obtained foot data were selected for further comparative analysis when a whole gait cycle on the force plates was properly identified. Two gait cycles per walk could be measured by the OMCS. If slippage of a step over the force plate was found on the video, that gait cycle was excluded from the analysis. Subjects were not informed about the necessity of proper foot placement on the force plates to avoid its potential influence on their gaits. All gait parameters of the limb calculated by Physilog® were comparatively analyzed with the same parameters calculated within the Visual3D pipeline (C-motion Inc., Germantown, MD, USA) using the OMCS data. Table 1 provides the definitions of each parameter based on the instruction manual provided with Physilog®. To measure the repeatability of the Physilog®, all subjects returned for a second day of testing within two weeks. The protocol remained identical to the first day, and the order of the trials was preserved.

**Table 1:** Definitions of gait parameters

Parameter	Unit	Definition
Cycle duration	Second (s)	The time between the heel touching the ground and the ipsilateral heel touching the ground again, meaning one gait cycle
Cadence	Steps per minute (steps/min)	The number of cycles in a minute
Stance phase (Stance)	Percent (%) of cycle duration	The percentage of time a part of the foot touches the ground during a gait cycle
Swing phase (Swing)	Percent (%) of cycle duration	The percentage of time the foot are in the air and not touching the ground during a gait cycle
Loading phase (Loading)	Percent (%) of stance	The percentage of time between the heel strike and the foot being flat on the ground during stance phase
Foot-flat phase (Foot-flat)	Percent (%) of stance	The percentage of time where the foot is fully flat on the ground during stance phase
Pushing phase (Pushing)	Percent (%) of stance	The percentage of time between the foot being flat on the ground and the toe leaving the ground at take-off during stance phase
Double support phase (Double support)	Percent (%) of cycle duration	The percentage of time both feet touches the ground during a gait cycle
Stride length	Meter (m)	The distance between two consecutive footprints on the ground, meaning the distance from the heel of one foot to the heel of the same foot one cycle later
Stride velocity	Meter per second (m/s)	The forward speed of one cycle
Peak angle velocity (Peak ang. velocity)	Degree per second (°/s)	The maximum angular velocity during the swing phase, between maximal heel clearance and minimal toe clearance
Maximal swing speed (Swing speed)	Meter per second (m/s)	The maximum forward speed of the foot during swing phase
Strike angle	Degree (°)	The angle between the foot and the ground at heel contact
Lift-off angle	Degree (°)	The angle between the foot and the ground at the end of the pushing phase
Swing width	Meter (m)	The maximal lateral excursion between the forward path and the real path of the foot during swing phase
3D path length	Percent (%) stride length	The length of the path of the foot during one cycle in 3D space
Maximal heel clearance (Max. heel)	Meter (m)	The maximal height above the ground reached by the heel during one cycle
Maximal toe clearance 1 (Max. toe1)	Meter (m)	The maximal height above the ground reached by the toe just after heel maximal clearance
Minimal toe clearance (Min. toe)	Meter (m)	The minimum height of the toe during swing phase
Maximal toe clearance 2 (Max. toe2)	Meter (m)	The maximal height above the ground reached by the toe just before heel contact

### WGAS

The gait analysis system of the Physilog® consisted of two small (50 mm × 37 mm × 9.2 mm), lightweight (19 g), inertial sensors for each foot, elastic straps to attach the sensors to the dorsum of the foot, and Gait Analyser software version 3.1 (GaitUp) running on a Windows PC (Microsoft, Redmond, WA, USA). Two sensors on both feet can be synchronized wirelessly, and no calibration procedure is required before and during the measurement. The algorithm estimates vertical alignment by detecting the vertical gravity axis from the accelerometer during the standing posture and azimuth alignment by maximizing the pitch angular velocity during walking. The position of the sensor on the foot does not affect the measurement [33]. Signals were sampled at 200 Hz and stored on an internal memory card. The recorded data were converted to left and right spatiotemporal gait parameters (per gait cycle) using the Gait Analyser software. The dedicated algorithms have been described elsewhere [24, 32–34]. For analysis, gait cycles in which all of the obtained parameters were error-free were used.

### OMCS

As a reference for the WGAS, lower extremity kinematic and kinetic data were measured by the system consisting of three-dimensional motion analysis and force platforms. A 12-camera system (Raptor-E, Motion Analysis Corp., Santa Rosa, CA, USA) captured the motion at a sample rate of 200 Hz, and four force platforms (AMTI, Watertown, MA, USA) recorded the ground reaction forces (GRF) at 1000 Hz. Twenty-three, 12.7-mm-diameter, retro-reflective markers were placed on specific locations of the pelvis, thighs, knees, lower legs, ankles, and feet to calculate joint centers and segment positions and to track segment motions, as mentioned above, followed by data processing with custom Visual3D software. The lower body pipeline, which is based on the Helen Hayes model, was used to calculate left and right spatiotemporal gait parameters. The data for the marker positions and force were smoothed by a fourth-order, zero phase shift, Butterworth low-pass filter at a cutoff frequency of 6 Hz for the positional data and 18 Hz for the force data. The cutoff frequency was determined by conducting a residual analysis. Initial contact and toe-off events were defined as when the vertical component of the unfiltered GRF exceeded and fell below 10 N, respectively.

**Fig. 2:**
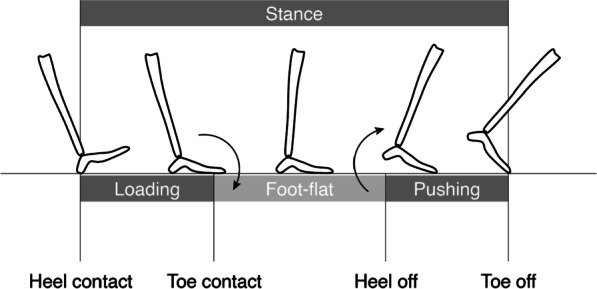
Temporal events during the stance phase. Toe contact and heel off events are defined as when the pitch angular velocity of the foot segment reaches above − 2 rad/s and below − 1 rad/s during the stance, respectively, and are used to distinguish among loading, foot-flat, and pushing in the stance phase

As the laboratory coordinate system, camera calibration was conducted just before the motion measurement. The Y_lab_ and Z_lab_ axes corresponded to the posterior-anterior (direction of travel is positive) and inferior-superior (vertical upward direction is positive) directions, respectively. The X_lab_ axis (with the medial–lateral direction, right side to the direction of travel is considered positive) was calculated from the external product of the Y_lab_ vector and Z_lab_ vectors. Participants were instructed to walk in the positive direction of the Y_lab_ axis. The segmental coordinate system of the foot segment was constructed from the markers affixed to the participants’ feet. For the right foot segment, the midpoint of the line segment connecting the medial and lateral malleoli of the ankle joint was set as the joint center of the ankle, and the vector from the medial malleolus to the lateral malleolus was set as the X_foot_ axis for the right foot segment (the vector from the lateral malleolus to the medial malleolus was set as the X_foot_ axis for the left foot segment). Next, the midpoint of the markers affixed to the distal ends of the first and fifth metatarsals was defined as the toe, and the Z_foot_ axis was defined as the external product of the X_foot_ vector and the vector from the joint center of the ankle to toe. The Y_foot_ axis was defined as the vector calculated by the external product of the Z_foot_ and X_foot_ vectors. To generate loading, foot-flat, and pushing (Fig. 2), the stance phase was separated based on the waveform data of the pitch angular velocity of the foot segment. The events of toe contact and heel off were defined by the pitch angular velocity of the foot segment being above − 2 rad/s and below − 1 rad/s during the stance, respectively [34].

### Statistical analysis

Statistical analysis was performed using SPSS version 23 (SPSS Inc., Chicago, IL, USA) and GraphPad Prism version 8.4.2 (GraphPad Software, San Diego, CA, USA). The distribution of each variable was checked for normality using the Anderson–Darling test. To compare the data of the same parameter obtained by the Physilog® and the OMCS, the absolute difference and Pearson’s correlation coefficients (r) were calculated. To visualize the amount and tendency of the system deviations of the two systems, a Bland–Altman plot was used. Limits of agreement were calculated as Mean_diff_WO_ ± (1.96 × SD_diff_WO_) with Mean_diff_WO_ being the mean difference between the WGAS and OMCS and SD_diff_WO_ being the standard deviation of the mean difference between the WGAS and OMCS. Lin’s concordance correlation coefficient (LCC), an index of how a new test reproduces a gold standard test, evaluates the degree to which pairs of observations fall on the 45° line through the origin [37]. To determine the repeatability of the WGAS measurements, LCC, r, and intraclass correlations [ICC(3,1)] between the two testing days were calculated. The following criteria were used to determine the strength of agreement for all parameters. The extent of LCC was categorized into 4 groups: Excellent (0.75–1.00), Good (0.60–0.74), Fair (0.40–0.59), and Poor (< 0.40) [38]. ICC(3,1) is a common statistic for evaluating repeatability [39] and is needed to calculate the minimally detectable change (MDC). SEM is the standard error of measurement, and SD is the standard deviation of the measure. SEM and MDC were calculated by the following formulas.

$$\begin{aligned} {\text{SEM}} & = {\text{SD}} \times \sqrt {1 - ICC} \\ {\text{MDC}} & = {\text{SEM}} \times 1.96 \times \sqrt 2 \\ \end{aligned}$$

## Results

**Fig. 3:**
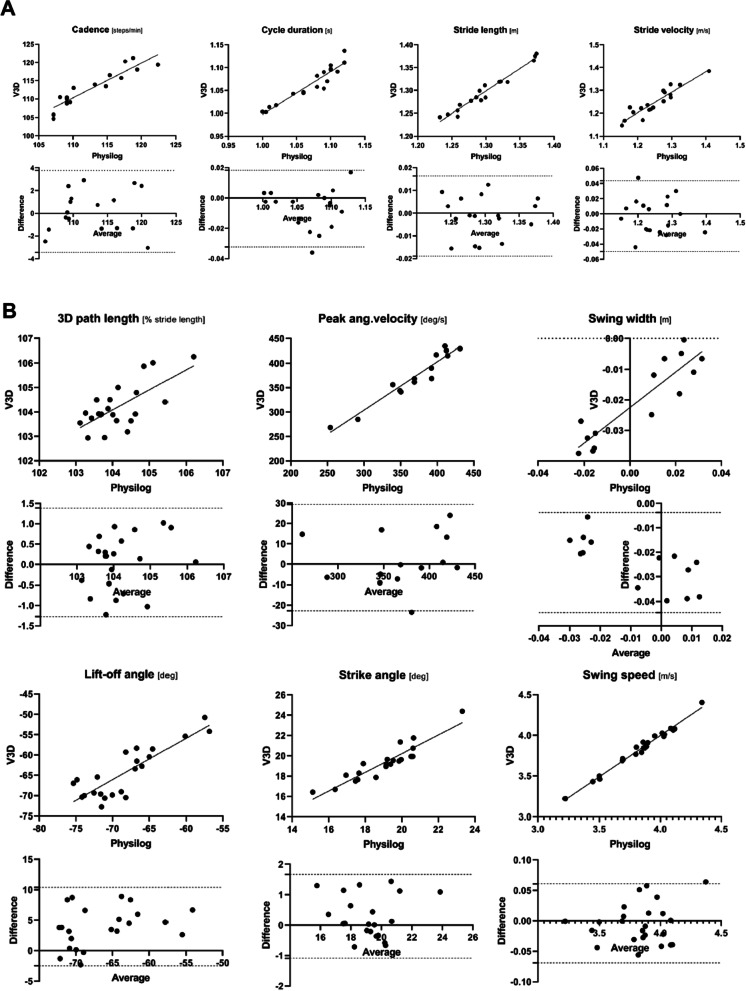
Scatter and Bland–Altman plots of the four general (**A**) and six spatial (**B**) gait parameters of the OMCS and the Physilog®. In the lower row, the Y-axis of the plot corresponds to the difference of the two measurement systems (OMCS minus Physilog®), whereas the X-axis is the average of the two measurements. Solid lines mark the average difference for the whole sample, and the dashed lines correspond to the 95% limits of agreement. 3D path length measures the actual length of the trajectory of the foot during one cycle in 3D space

**Fig. 4:**
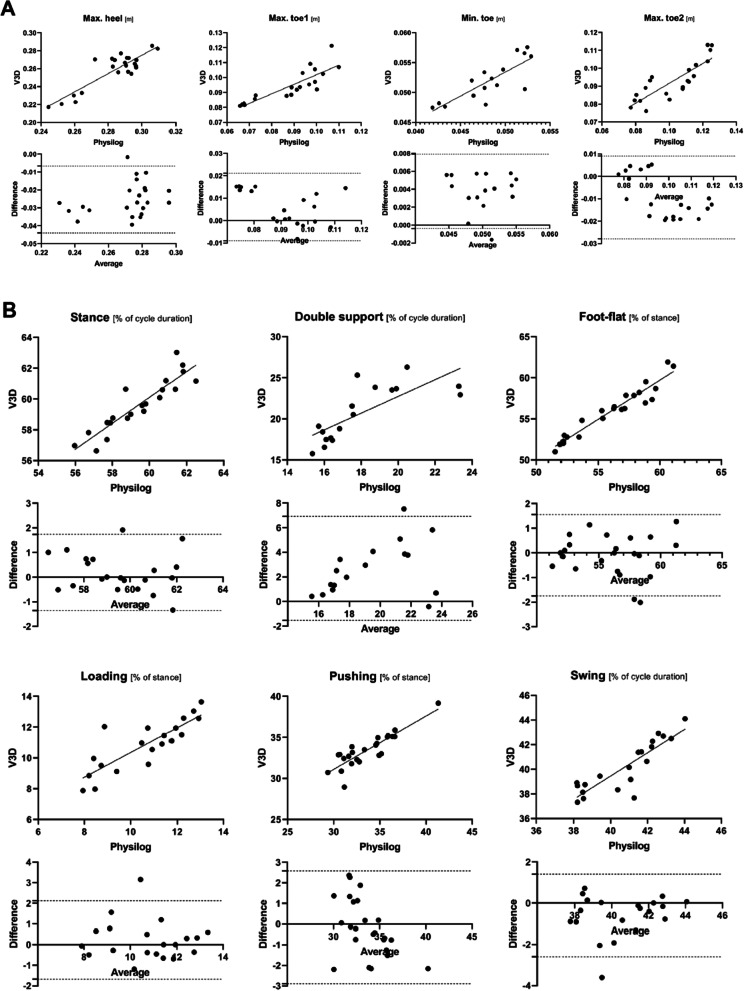
Scatter and Bland–Altman plots of the clearance (**A**) and temporal (**B**) gait parameter for validity of both Visual3D (V3D) and Physilog® configurations during overground walking conditions. In the lower row the Y-axis of the plot corresponds to the difference between the two measurement systems (V3D minus Physilog®), while the X-axis is the average of the two measurements. Solid lines mark the average difference for the whole sample, while the dashed lines correspond to the 95% limits of agreement. Max. toe1 means the maximal height above the ground reached by the toe just after heel maximal clearance and Max. toe2 the maximal height above the ground reached by the toe just before heel contact. The values are based on the result of the left foot of the patient. The stance phase is defined as the period of time where the foot is in contact with the ground

Two hundred and thirty-eight gait cycles were simultaneously obtained with the WGAS and the OMCS. Among them, 214 gait cycles were discarded, because of improper foot contact with the force plates and errors to generate a full set of the WGAS parameters. Twenty-four gait cycles were consequently selected for further analysis. The statistics for validity of all gait parameters calculated with the WGAS are summarized in Table 2. Bland–Altman plots of all parameters are also shown in Figs. 3 and 4. The WGAS demonstrated excellent validity in 12 of 20 parameters, including all general gait parameters (cadence, cycle duration, stride length, and stride velocity), most temporal gait parameters (stance, foot-flat, loading, pushing, and swing), and several spatial parameters (strike angle, swing speed, and peak angular velocity) (Table 2). Good validity was found for 2 spatial parameters (3D path length and lift-off angle) and 2 feet clearance parameters (maximum toe 1 and 2). Four parameters (swing width, maximum heel, minimum toe, and double support) were identified as fair, but all of them had high Pearson’s correlation coefficients ranging from 0.76 to 0.9. The results of repeatability are summarized in Table 2. The WGAS demonstrated excellent repeatability when measured between sessions (0.70 < LCC < 0.96). This was also the case when using other repeatability metrics (0.82 < r < 0.96 and 0.83 < ICC (3,1) < 0.98).

**Table 2:** Statistical parameters of validity and repeatability of all gait parameters calculated with Physilog®

				95% Limits of agreement		
Validity	LCC	r	Bias	Lower bound	Upper bound	Significance
*General*						
Cadence	0.930	0.931	0.169	− 3.440	3.777	< 0.0001
Cycle duration	0.932	0.947	− 0.007	− 0.032	0.018	< 0.0001
Stride length	0.979	0.979	− 0.001	− 0.019	0.016	< 0.0001
Stride velocity	0.927	0.929	− 0.003	− 0.050	0.044	< 0.0001
*Spatial*						
3D path length	0.676	0.688	0.058	− 1.271	1.386	0.0004
Peak ang. Velocity	0.961	0.964	3.320	− 22.72	29.36	< 0.0001
Swing width	0.406	0.903	− 0.024	− 0.045	− 0.004	< 0.0001
Lift-off angle	0.674	0.852	3.947	− 2.481	10.37	< 0.0001
Strike angle	0.912	0.925	0.290	− 1.086	1.665	< 0.0001
Swing speed	0.991	0.992	− 0.004	− 0.069	0.061	< 0.0001
*Clearance*						
Max. heel	0.438	0.877	− 0.025	− 0.044	− 0.007	< 0.0001
Max. toe1	0.729	0.850	0.006	− 0.009	0.021	< 0.0001
Min. toe	0.506	0.817	0.004	− 0.000	0.008	< 0.0001
Max. toe2	0.624	0.840	− 0.009	− 0.028	0.009	< 0.0001
*Temporal*						
Stance	0.898	0.906	0.191	− 1.360	1.741	< 0.0001
Double support	0.509	0.764	2.700	− 1.532	6.933	0.0004
Foot-flat	0.961	0.962	− 0.099	− 1.748	1.550	< 0.0001
Loading	0.827	0.836	0.226	− 1.671	2.122	< 0.0001
Pushing	0.832	0.869	− 0.155	− 2.895	2.585	< 0.0001
Swing	0.829	0.871	− 0.593	− 2.591	1.406	< 0.0001

Repeatability	LCC	r	ICC (3, 1)	MDC	Significance
*General*					
Cadence	0.955	0.960	0.977	1.902	< 0.0001
Cycle duration	0.958	0.964	0.979	0.017	< 0.0001
Stride length	0.851	0.861	0.921	0.051	0.0014
Stride velocity	0.763	0.824	0.869	0.088	0.0034
*Spatial*					
3D path length	0.890	0.975	0.943	0.292	< 0.0001
Peak ang. Velocity	0.944	0.946	0.971	23.483	< 0.0001
Swing width	0.880	0.902	0.944	0.011	0.0004
Lift-off angle	0.938	0.939	0.968	3.534	< 0.0001
Strike angle	0.899	0.900	0.947	1.409	0.0004
Swing speed	0.861	0.874	0.928	0.191	0.001
*Clearance*					
Max. heel	0.852	0.872	0.921	0.015	0.001
Max. toe1	0.899	0.934	0.949	0.009	< 0.0001
Min. toe	0.836	0.842	0.912	0.005	0.0023
Max. toe2	0.954	0.957	0.978	0.008	< 0.0001
*Temporal*					
Stance	0.836	0.893	0.942	0.824	0.0005
Double support	0.703	0.845	0.838	1.729	0.0021
Foot-flat	0.894	0.901	0.944	2.608	0.0004
Loading	0.854	0.863	0.922	1.054	< 0.0001
Pushing	0.896	0.913	0.945	2.267	0.0002
Swing	0.836	0.893	0.942	0.824	0.0005

## Discussion

The present study conducted a comprehensive validation of the spatiotemporal gait parameters Physilog® calculates in healthy individuals and demonstrated that they were reliably accurate and repeatable. In particular, the general and temporal gait parameters showed excellent accuracy and reproducibility. The spatial and clearance gait parameters were good or fair in LCCs, but they had high correlation coefficients with the OMCS and great reproducibility. These results provide the scientific basis for conducting clinical gait analysis with the WGAS for healthy subjects.

Since the WGAS consists of an accelerometer and a gyroscope, it is highly likely that the gait parameters directly calculated from their raw values are valid. In fact, Peak ang. Velocity, raw data of the accelerometer, showed a high degree of agreement with the OMCS. On the other hand, most gait parameters depend on the information of temporal events and trajectories the algorithm assumes, such as the timing of grounding and take-off of feet. Therefore, the accuracy of most gait parameters relies totally on the accuracy of the estimate the algorithm generates. The calculation of the general and temporal gait parameters showed excellent validity except for double support, even though it requires the information for the timing of initial contact and toe-off of one foot, indicating the high accuracy of the estimate for the timing of temporal foot events by Physilog®. This is in contrast with the previous validation study of Physilog® in hemiplegic patients revealing low accuracy of its temporal gait parameters due to inadequate detection of the timings of initial contact and toe-off [22]. Unlike other temporal gait parameters, the validity of double support was not very good. It is because its calculation needs the information for the timing of initial contact and toe-off of both feet [21]. The calculation of the foot clearance parameters is more challenging than for other parameters, since it requires more gait event information, such as timings of heel striking and toe off, and the orientation and trajectory of the foot [33]. Accordingly, the LCCs of Max heel and Min toe were found to be fair. But their correlation coefficients were high, suggesting its usability for clinical gait studies. Based on these results, it can be concluded that the calculation of the gait parameters by the WGAS is reasonable.

Regarding the reproducibility of the WGAS, there was a potential concern, because it does not have precisely defined rules for placing, holding, and synchronizing the sensors [40, 41]. In fact, the present study demonstrated that Physilog® had excellent repeatability, indicating that the WGAS retains robust accuracy without losing its ease of use. When comparing the WGAS to another versatile gait analysis method such as conventional visual observations, the WGAS is apparently more quantitative, accurate, and reproducible. Collectively, it can be concluded that the WGAS is reasonably usable for clinical gait analysis.

Although the present study shows the great potential of the WGAS for clinical gait analysis, to expand its application, the limits within which the WGAS can provide accurate measurements need to be determined. Because its algorithm determines the stance phase based on the differential value of the normal foot pitch angular velocity and foot angular velocity, in the case of abnormal gaits such as drop foot and circumduction gait, it is likely that the algorithm fails to provide accurate estimates. Therefore, to expand the application of the WGAS to patients with gait disorders, a study similar to the present one but including a wide range of participants with different gait disorders needs to be conducted.

The current study has several limitations. Most importantly, the number of subjects is too small to generalize the obtained findings about the WGAS. Further, female subjects need to be analyzed, since sex difference affects gait characteristics [42, 43]. A large cohort study consisting of people with diverse types of backgrounds will clarify the reliability and usability of the WGAS. Although the number of subjects is small in the present study, the obtained results could be a reasonable basis to perform the validation study in a large number of people.

The WGAS is a fascinating tool that allows gait analysis even under daily living conditions outside of the laboratory. Potentially, it could collect gait data before and during a falling event, detect subtle gait disturbances that physicians cannot notice in the clinic, or screen subjects for detailed OMCS analysis. The present study contributes to such practical use of the WGAS by providing the evidence that the WGAS fulfills the requirements for a clinical gait analysis tool.

## Conclusions

The present study showed that spatiotemporal gait parameters estimated by Physilog® were reasonably accurate and repeatable when used in healthy young adults, providing a scientific basis for applying this system to clinical studies. Because the WGAS is usable both inside and outside of the laboratory and has great versatility, it could open a new era of gait analysis research by analyzing many subjects in different situations. To expand its application to patients presenting with gait disorders, further study is necessary to clarify the range that Physilog® can analyze accurately.

## Data Availability

The datasets used and/or analysed during the current study are available from the corresponding author on reasonable request.

## Abbreviations

WGAS: Wearable gait analysis system
OMCS: Optical motion capture system
GRF: Ground reaction forces
LCC: Lin’s concordance correlation coefficient
MDC: Minimally detectable change
